# A Study of Clinical Profile and Factors Affecting Mortality in Patients With Acute-on-Chronic Liver Failure in a Tertiary Care Hospital in Nepal

**DOI:** 10.7759/cureus.86468

**Published:** 2025-06-20

**Authors:** Pradip Kumar Kafle, Anurag Jha, Brindeswari Kafle Bhandari, Rabin Hamal, Tshering W Sherpa, Dinesh Koirala, Tulsiram Bhattarai, Manoj Lamsal, Sushil Parajuli, Rahul Pathak

**Affiliations:** 1 Gastroenterology, Tribhuvan University Institute of Medicine, Kathmandu, NPL; 2 Medicine, Kathmandu Medical College and Teaching Hospital, Kathmandu, NPL

**Keywords:** aarc score, aclf, acute-on-chronic liver failure, clif-c aclf, easl-clif

## Abstract

Background and aim

Acute-on-chronic liver failure (ACLF) is a dynamic condition with very high short-term mortality. Although several mortality predictors have been studied, no single factor can reliably predict its course and outcome. This study aimed to evaluate various biochemical parameters and severity scores at presentation to assess their ability to predict mortality in ACLF patients.

Methods

An observational cross-sectional study was conducted from March 2024 to February 2025 at a tertiary care center in Nepal. A total of 51 patients were enrolled over one year. ACLF was diagnosed based on the Asia-Pacific Association for the Study of the Liver (APASL) criteria and/or the European Association for the Study of the Liver-Chronic Liver Failure Consortium (EASL-CLIF) criteria. Clinical features, biochemical parameters, and severity scores at admission were analyzed.

Results

The overall mortality rate was high (56.9%), consistent with previous studies. About 28% of patients who met the APASL definition of ACLF did not fulfill the EASL-CLIF criteria. On univariate analysis, factors such as oliguria, hepatic encephalopathy (HE) grade, ACLF grade, total serum protein, urea, ascitic fluid total leukocyte count, serum lactate, and APASL-ACLF Research Consortium (AARC) score were associated with mortality. However, multivariate analysis did not identify a single independent predictor of mortality. Different scoring systems were useful in predicting mortality at admission. Among them, the CLIF-C ACLF score had the highest predictive accuracy (area under the curve (AUC): 0.990), followed by the AARC score (AUC: 0.79), Sequential Organ Failure Assessment (SOFA) score, and Model for End-Stage Liver Disease (MELD) score. However, the Child-Turcotte-Pugh (CTP) score at admission was not effective in predicting mortality.

Conclusion

ACLF represents an acute flare of inflammation on a background of chronic liver disease. Using multiple mortality predictors at the time of admission can help guide treatment decisions and provide better prognostication of patient outcomes.

## Introduction

Acute deterioration of chronic liver disease is a defining feature of acute-on-chronic liver failure (ACLF). The Asia-Pacific Association for the Study of the Liver (APASL) defines ACLF as acute hepatic insult manifesting as jaundice and coagulopathy complicated within four weeks by ascites and/or encephalopathy in a patient with previously diagnosed or undiagnosed chronic liver disease with high short-term mortality [1].

In contrast, it is described as an acute deterioration of pre-existing, chronic liver disease, usually related to a precipitating event and associated with increased mortality at three months due to multi-system organ failure by the working groups of the American Association for the Study of Liver Diseases (AASLD) and the European Association for the Study of the Liver (EASL) [2,3]. Liver injury is the primary component in the APASL statement, whereas organ failure is the main component of ACLF according to the EASL-AASLD consensus. This new entity was necessary because, if the triggering event can be controlled, ACLF may be reversible; if not, the short-term death rate is significant, ranging from 50% to 90% [2-7]. Infections like chronic hepatitis B reactivation are common in the East, whereas alcohol and bacterial infections are typically the acute insult in the West [1,8]. There is a wide range of data from India, and research has found that non-hepatic insults and hepatitis B reactivation are frequent acute precipitant variables [9,10]. This demonstrates that there are etiological disparities within the same country as well as between its various centers.

Because the two groups' definitions of ACLF differ fundamentally, data from the APASL and the EASL collaboration have produced a range of outcomes [8]. A new CLIF-C ACLF score system has been developed by the EASL consortium to better prognosticate these patients and determine which ones require liver transplantation right away. Similar studies have been done from the Indian subcontinent as well as by the Indian National Association for the Study of the Liver (INASL) consortium, which involved retrospective data and supported the fact that organ failure predicts outcome [9-11]. Some new simplified scores have been developed [12]. Since the study on ACLF has not been carried out extensively in Nepal, our current study aimed to identify the acute insults in ACLF and to identify predictors of in-hospital mortality in these patients.

## Materials and methods

Study method

This is a single-centered observational cross-sectional study.

Study population

Patients with ACLF who met the inclusion criteria were included in the study from March 2024 to February 2025.

Study site

The study was done in the Department of Gastroenterology, Tribhuvan University Institute of Medicine, Kathmandu, Nepal.

Sample size

The minimum required sample size was calculated by using the formula $\text{sample size}=\text{Z2}&times;\frac{\text{pq}}{\text{e2}}$ where p is the incidence of ACLF in cirrhotic patients in Nepal and is equal to 3% [1], q=1-p, e is the precision and is equal to 0.05, Z is the confidence interval and is equal to 1.96, and the sample size is equal to 51.

Sampling method

Non-probability method was used to include the patients in the study.

Inclusion and exclusion criteria

Patients diagnosed with ACLF based on APASL and/or EASL-Chronic Liver Failure Consortium (CLIF) criteria and aged between 18 and 79 years were included in the study. However, patients diagnosed with ACLF based on criteria other than APASL and/or EASL-CLIF were excluded from the study. Similarly, patients fulfilling the APASL and or EASL-CLIF criteria with age <18 years, who were pregnant, with portal vein thrombosis, with hepatocellular carcinoma, and who were unwilling to participate in the study were also excluded. 

Data collection tools and variables

Data was collected in a proforma. We collected data based on history, examination findings, and laboratory investigations. Chronic liver disease and ACLF were defined based on history, clinical examination, and laboratory investigations. The variables included in the study were sociodemographic variables like age and sex, comorbidities, laboratory investigation parameters, and several scoring systems for ACLF. The scoring systems were Child-Turcotte-Pugh (CTP), Model for End-Stage Liver Disease (MELD), Sequential Organ Failure Assessment (SOFA), APASL-ACLF Research Consortium (AARC), and CLIF-C ACLF [13-15]. These scoring systems were calculated manually and using mobile apps. The outcomes of the study were mortality and duration of hospitalization.

Ethics

The Institutional Review Committee of Tribhuvan University Institute of Medicine granted ethical approval (approval number: 517(6-11) E2 080/081). All study participants gave their informed consent to allow the use of anonymous personal and clinical data in research, which was used to ensure complete confidentiality.

Statistical analysis

Data was compiled, edited, and checked daily to maintain consistency. The data was collected in Microsoft Excel (Microsoft Corporation, Redmond, Washington, United States). For statistical analysis, IBM SPSS Statistics for Windows Version 27.0 (Released 2019; IBM Corp., Armonk, New York, United States) was used. Quantitative variables are expressed as mean±SD and categorical variables as frequency and percentage. The normally distributed continuous and categorical variables were compared using Student's t-test and the chi-squared test, respectively. Univariate and multivariate logistic regression analyses with odds ratios were done to ascertain the predictors of mortality. Mortality prediction of various scores is compared by receiver operating characteristic (ROC) curve analysis by calculating the area under the curve (AUC). A p-value of <0.05 was considered statistically significant.

## Results

A total of 51 patients who fulfilled our inclusion and exclusion criteria were included in the study. The mean age of presentation was 45.76±11.81 years. In our study, 37 (72.5%) were male, while 14 (27.5%) were female. The most common clinical symptoms were jaundice (51, 100%), followed by abdominal distension (50, 98%), pedal edema (46, 90.2%), and oliguria (39, 76.5%). Icterus and ascites were present in all patients. Alcohol is the most common cause of cirrhosis, which is present in 36 (70.6%) patients, followed by chronic hepatitis B (8, 15.7%), non-alcoholic steatohepatitis (NASH) (2, 3.9%), chronic hepatitis C (2, 3.9%), and Wilson disease (1, 2%). Drug-induced liver injury (DILI) (in the form of complementary and alternative medicine (CAM)) was the most common precipitating insult in 15 (29.4%) patients. The demographic profile, clinical features, examination findings, and laboratory investigation are shown in Table 1.

**Table 1:** Baseline parameters between dead and survived on admission CAM: complementary and alternative medicine; HE: hepatic encephalopathy; CLD: chronic liver disease; ALD: alcoholic liver disease;  NASH: non-alcoholic steatohepatitis; ACLF: acute-on-chronic liver failure; DILI: drug-induced liver injury; APASL: Asia-Pacific Association for the Study of the Liver; EASL: European Association for the Study of the Liver; CLIF-C ACLF: Chronic Liver Failure Consortium acute-on-chronic liver failure; AARC: APASL-ACLF Research Consortium; TLC: total leukocyte count; AST: aspartate transaminase; ALT: alanine transaminase; ALP: alkaline phosphatase; PT: prothrombin time; INR: international normalized ratio; ABG: arterial blood gas

	Dead	Survived	P-value
Gender			
Male	20 (69)	17 (77.3)	0.510
Female	9 (31)	5 (22.7)	
Clinical symptoms			
Jaundice	29	22	
Abdominal distension	29	21 (95.5)	0.246
Pedal edema	28 (96.6)	18 (81.8)	0.080
Oliguria	26 (89.7)	13 (59.1)	0.011
CAM	19 (65.5)	16 (72.7)	0.583
Past history of jaundice	21 (72.4)	10 (45.5)	0.051
Altered sensorium	25 (86.2)	14 (63.6)	0.060
Hematemesis	9 (31)	2 (9.1)	0.059
Melena	11 (37.9)	5 (22.7)	0.246
Blood transfusion	11 (37.9)	2 (9.1)	0.019
HE			
Absent	1 (3.4)	8 (36.4)	0.016
Grade 1	8 (27.6)	7 (31.8)	
Grade 2	10 (34.5)	5 (22.7)	
Grade 3	7 (24.1)	2 (9.1)	
Grade 4	3 (10.3)	0	
Etiology of CLD			
ALD	19 (65.5)	17 (77.3)	0.746
Hepatitis B	5 (17.2)	3 (13.6)	
Hepatitis C	1 (3.4)	1 (4.5)	
NASH	2 (6.9)	0	
Wilson disease	1 (3.4)	0	
Others	1 (3.4)	1 (4.5)	
Precipitating factor of ACLF			
Alcohol	3 (10.3)	6 (27.3)	0.406
Hepatitis A	3 (10.3)	1 (4.5)	
Hepatitis E	2 (6.9)	1 (4.5)	
Acute hepatitis B reactivation	2 (6.9)	0	
DILI	10 (34.5)	5 (22.7)	
Alcohol+DILI	5 (17.2)	7 (31.8)	
Others	4 (13.8)	2 (9.1)	
APASL criteria	29 (100)	21 (95.9)	0.246
EASL criteria	21 (75)	15 (75)	1.000
Grade of CLIF-C ACLF			
Ia	0	5 (33.3)	<0.001
Ib	0	2 (13.3)	
II	5 (23.8)	8 (53.3)	
IIIa	15 (71.4)	0	
IIIb	1 (4.8)	0	
Grade of AARC score			
1	0	0	<0.001
2	2 (6.9)	11 (52.4)	
3	27 (93.1)	10 (47.6)	
Age	47.83±11.86	43.05±11.44	0.154
Duration of stays (in days)	11.58±8.58	15.09±5.78	0.105
Amount of alcohol (in grams)	159.58±69.99	148.42±59.09	0.582
Duration of alcohol (in years)	11.41±4.53	12.26±4.65	0.574
Ascitic fluid TLC	916.60±931.30	408.00±363.90	0.025
Urea (mmol/l)	23.34±19.20	8.47±5.91	0.023
Creatinine (mmol/l)	246.24±148.89	184.68±156.40	0.159
Total bilirubin	403.66±187.57	391.81±158.52	0.813
Direct bilirubin	217.68±108.92	191.81±76.05	0.346
Indirect bilirubin	204.71±94.71	194.86±92.77	0.712
AST	213.48±120.38	195.36±119.82	0.729
ALT	106.10±12.34	94.95±80.67	0.816
ALP	132.05±84.27	133.95±38.68	0.922
Total serum protein	59.93±19.32	54.86±13.66	0.300
Serum albumin	20.67±6.25	21.82±8.20	0.568
PT	38.20±12.15	35.52±13.51	0.460
INR	5.05±3.99	4.21±4.05	0.465
ABG pH	6.75±1.57	6.99±1.14	0.552
ABG lactate	3.28±2.46	1.61±0.61	0.031

Out of 51 patients, 29 (56.9%) died. About 50 (98%) patients followed the APASL definition of ACLF; however, only 36 (70.6%) met the EASL definition of ACLF. While staging and comparing mortality ACLF based on AARC score, we found that no one in grade 1 died and 27 (93.1%) patients in stage 3 died. While comparing similar findings in patients who met EASL-CLIF, no patients survived.

Mortality predictors

On univariate analysis, patients with a decrease in urine output, who needed multiple blood transfusions, and who had high HE grade, large varices in endoscopy, higher grades of ACLF, low total serum protein, high urea level, high ascitic total count, and high lactate on admission day died more and were statistically significant (p<0.05 for every variable). Multivariate analysis of those variables was not able to show statistical significance in predicting mortality (Table 2).

**Table 2:** Predictors of mortality in multivariate analysis HE: hepatic encephalopathy; EASL-ACLF: European Association for the Study of the Liver acute-on-chronic liver failure; TLC: total leukocyte count; ABG: arterial blood gas; AARC: APASL-ACLF Research Consortium

	SE	P-value	B
Oliguria	49954.911	0.999	0.000
Blood transfusion	13115.798	0.980	0.000
HE grade	1284.197	0.983	0.000
Endoscopy	5461.068	0.998	0.000
Grade of EASL-ACLF	23321.765	0.974	0.000
Total serum protein	57.119	0.981	0.258
Urea	239.249	0.991	15.042
Ascitic fluid TLC	5.946	0.975	1.207
ABG lactate	1791.559	0.993	12913906.435
AARC score	3747.933	0.997	0.000

**Figure 1:**
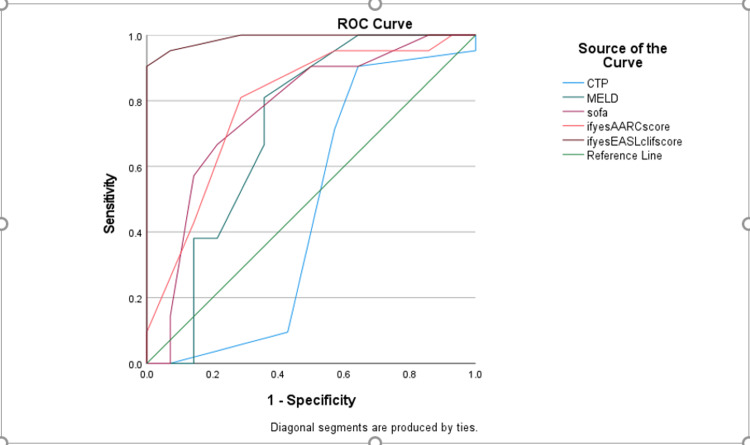
ROC curves of scores predicting mortality on admission ROC: receiver operating characteristic; CTP: Child-Turcotte-Pugh; MELD: Model for End-Stage Liver Disease; SOFA: Sequential Organ Failure Assessment; AARC: APASL-ACLF Research Consortium

We compared the predictability of each score with mortality using the ROC curve, calculating the AUC for each component. CLIF-C ACLF has predictability with an AUC of 0.990, followed by AARC with an AUC of 0.791, SOFA with an AUC of 0.770, and MELD with an AUC of 0.718 (Table 3); however, the CTP score with an AUC of 0.464 did not show concordance to predict mortality (Figure 1).

**Table 3:** Results from the AUC for scores predicting mortality on admission AUC: area under the curve; CTP: Child-Turcotte-Pugh; MELD: Model for End-Stage Liver Disease; SOFA: Sequential Organ Failure Assessment; AARC: APASL-ACLF Research Consortium; CLIF-C ACLF: Chronic Liver Failure Consortium acute-on-chronic liver failure

Test result variable(s)	Area	SE	Asymptotic sig.	Asymptotic 95% CI		Off	Sensitivity	Specificity
				Lower bound	Upper bound			
CTP	0.464	0.116	0.724	0.237	0.692	11	66.7	66.7
MELD	0.718	0.101	0.031	0.520	0.916	24	56.86	56.86
SOFA	0.770	0.087	0.007	0.599	0.942	5	60.6	88.9
AARC score	0.791	0.082	0.004	0.630	0.951	10	84.5	71
CLIF-C ACLF	0.990	0.012	<0.001	0.967	1.000	9	56.86	56.86

## Discussion

Since ACLF is a relatively new entity, our work contributes to the body of emerging and not yet comprehensive literature and evidence. When compared to the West, the etiology of both acute and chronic insults differs in the East [11,13,16-19]. The etiology of chronic liver disease varies greatly within the Indian subcontinent alone [13].

In 70.6% of the patients in our study, alcohol-related liver cirrhosis was the most common primary cause, followed by hepatitis B (15.7%). Although the results are consistent with a small number of other research, these findings contribute to the more varied nature of the underlying causes of cirrhosis [9,16,19] but contrast with other Indian research that found that only 10-30% of people had alcoholic cirrhosis [10,18,20]. According to certain studies, 30-64% of people with ACLF had cirrhosis, with chronic hepatitis B being the most common cause. Similarly, the majority of data from Taiwan and China likewise showed that hepatitis B virus (HBV) was the main cause of cirrhosis in individuals with ACLF [21,22]. However, 50% of patients in a big research by Moreau et al. had liver illness linked to alcohol in the West [8]. This demonstrates that there are etiological distinctions between the East and West of the planet.

In our study, 51 patients were enrolled, with an average age of presentation of 45.76±11.81 years. Males comprised 72.5% of the study population, indicating a male predominance. These findings are comparable to previous studies from India. Sarin et al. [13] reported an average age of 44.7±12 years with 82% male predominance, while Dhiman et al. [23] observed an average age of 46±13 years, with males accounting for 86% of cases. However, the CANONIC study [24] reported a slightly older average age of presentation (56.2±11.6 years), though males remained the predominant group (64.6%). The younger age of presentation in our study may be attributed to the etiology of cirrhosis. A study by Ferreira Cardoso et al. [25] found that ACLF in younger patients was predominantly associated with alcohol-related cirrhosis. Similarly, Arroyo et al. [26] reported ACLF occurring in relatively younger patients, particularly in those with alcohol-related liver disease and untreated chronic hepatitis B infection.

Regarding the etiology of cirrhosis, alcohol was the leading cause in our study, accounting for 70.6% of cases. This finding aligns with a previous Nepalese study, where alcohol-related cirrhosis constituted 84.94% of cases [27]. The high prevalence of alcohol consumption in Nepal could be attributed to factors such as easy availability, cultural acceptance, and inadequate governmental regulations. A study by Rathod et al. [28] reported that 15-57% of Nepali adults had consumed alcohol at some point, with 1.5-25% meeting the criteria for alcohol use disorder. In contrast, Nepal has a relatively low prevalence of hepatitis B (0.9%) and hepatitis C (0.38%) infections [29]. Although NASH is emerging as a significant etiology worldwide, the relatively low average BMI of our study population (20.9±1.8 kg/m²) suggests that it is not yet a major contributor in our setting [30].

DILI, primarily due to the consumption of CAM, was identified as the most common precipitating factor (29.4%), followed by concurrent use of alcohol and CAM and alcoholic hepatitis. Acute viral hepatitis, including hepatitis B reactivation, was responsible for only 18% of cases. The use of herbal products is widespread in Nepalese society, often preceding disease exacerbation and hospitalization. Although there is limited local data, a study from India by Philips et al. [31] found that 32% of patients had used CAM before being diagnosed with cirrhosis and 68% had used it at some point after diagnosis. Additionally, 39% of ACLF cases in that study were linked to CAM use.

The precipitating factors for ACLF vary across different regions. The study by Sarin et al. [13] in India identified alcohol (35.7%) as the most common precipitating factor, followed by viral hepatitis (21.4%) and sepsis (16.6%). In contrast, an Egyptian study by Zakareya et al. [32] found sepsis (49.8%) to be the leading precipitant. Similarly, a study from Taiwan by Huang et al. [33] reported reactivation of hepatitis B (68.4%) as the most common cause. The CANONIC study identified sepsis (32.6%) as the predominant precipitant [34]. Unlike these international studies, our study observed that sepsis and viral hepatitis, including hepatitis B reactivation, were minor contributors to ACLF.

ACLF is associated with high short-term mortality due to rapid hepatic decompensation and subsequent multi-organ failure. In our study, the overall mortality rate was 56.9%, occurring within two weeks of hospital admission. This finding is comparable to the study by Valantine et al. [35] in India, which reported a mortality rate of 56%. However, the CANONIC study [34] observed a lower mortality rate of 33%. The higher mortality rate in Asian populations may be attributed to the lack of or inadequate liver transplant facilities and the unavailability of dedicated liver intensive care units.

Univariate analysis in our study identified oliguria, HE grade, ACLF grades, total serum protein, urea levels, ascitic fluid total leukocyte count, serum lactate, and AARC score as significant predictors of mortality. These findings align with the study by Valantine et al. [35], where HE, total serum protein, and ascites were identified as mortality predictors. However, multivariate analysis in our study did not reveal any statistically significant predictors of mortality. This contrasts with the study by Valantine et al. [35], which found bilirubin levels to be a significant predictor. One limitation of our study was that we only assessed baseline parameters at admission without follow-up measurements. Evaluating these parameters at different time points (e.g., day 3, day 7) might have provided more conclusive insights.

Among various scoring systems, the CLIF-C ACLF score emerged as the most efficient predictor of mortality in our study, with an AUC of 0.99, followed by AARC (0.791), SOFA (0.770), MELD (0.710), and CTP (0.464). These findings are consistent with previous studies. Barosa et al. [36] demonstrated that the CLIF-C ACLF score was superior to SOFA, MELD, and CTP in predicting mortality. Similarly, Leão et al. [37] compared CLIF-C ACLF and AARC scores and found CLIF-C ACLF to be the better predictor. MELD was found to be a better predictor than CTP, as shown in the study by Antunes et al. [38]. The superior predictive ability of CLIF-C ACLF, AARC, and SOFA scores can be attributed to their inclusion of organ failure as a scoring component. In contrast, the CTP score primarily assesses hepatic dysfunction, making it a weaker predictor. The MELD score, which incorporates kidney function along with hepatic dysfunction parameters, is therefore a better predictor than CTP.

This study has some limitations. The sample size was small, which made it difficult to analyze subgroups based on regional differences. Mortality prediction was based on a single assessment of risk factors. Long-term follow-up was not included. Repeated measurements over time could have given better insights into outcomes, especially in critically ill ACLF patients.

## Conclusions

The short-term mortality rate of ACLF, an emergent entity, is extremely high. Both the acute triggering cause and the underlying chronic cause vary greatly throughout the world, including within Nepal. In order to classify patients with ACLF into limited intensive care units and develop new treatments, different prognostic ratings may be used. In forecasting these patients' short-term mortality, the CLIF-C ACLF scoring system outperforms conventional prognostic ratings. More research is needed to shed more light on this developing idea of ACLF and help us manage these patients more effectively.

## References

[1] Choudhury A, Kulkarni AV, Arora V (2025). Acute-on-chronic liver failure (ACLF): the 'Kyoto Consensus'-steps from Asia. Hepatol Int.

[2] Jalan R, Gines P, Olson JC (2012). Acute-on chronic liver failure. J Hepatol.

[3] Olson JC, Kamath PS (2011). Acute-on-chronic liver failure: concept, natural history, and prognosis. Curr Opin Crit Care.

[4] Laleman W, Verbeke L, Meersseman P (2011). Acute-on-chronic liver failure: current concepts on definition, pathogenesis, clinical manifestations and potential therapeutic interventions. Expert Rev Gastroenterol Hepatol.

[5] Chan AC, Fan ST, Lo CM (2009). Liver transplantation for acute-on-chronic liver failure. Hepatol Int.

[6] Mikolasevic I, Milic S, Radic M, Orlic L, Bagic Z, Stimac D (2015). Clinical profile, natural history, and predictors of mortality in patients with acute-on-chronic liver failure (ACLF). Wien Klin Wochenschr.

[7] Mikolasević I, Radić M, Milić S, Stimac D (2013). Acute-on-chronic liver failure (ACLF)--a new entity in hepatology? [Article in Croatian]. Lijec Vjesn.

[8] (2025). Diagnosis, prevalence, and prognosis of acute-on-chronic liver failure (ACLF) : results of the EASL-Chronic Liver Failure (CLIF) consortium : CANONIC study. https://difusion.ulb.ac.be/vufind/Record/ULB-DIPOT:oai:dipot.ulb.ac.be:2013/146181/Details.

[9] Duseja A, Chawla YK, Dhiman RK, Kumar A, Choudhary N, Taneja S (2010). Non-hepatic insults are common acute precipitants in patients with acute on chronic liver failure (ACLF). Dig Dis Sci.

[10] Garg H, Kumar A, Garg V, Sharma P, Sharma BC, Sarin SK (2012). Clinical profile and predictors of mortality in patients of acute-on-chronic liver failure. Dig Liver Dis.

[11] Jha AK, Nijhawan S, Rai RR, Nepalia S, Jain P, Suchismita A (2013). Etiology, clinical profile, and inhospital mortality of acute-on-chronic liver failure: a prospective study. Indian J Gastroenterol.

[12] Patwa AK, Yadav K, Atam V (2024). Comparison of a novel score "NOD-ACLF" to other established prognostic scores for prediction of mortality in APASL-ACLF patients: a cohort study from a tertiary care center of North India. J Clin Exp Hepatol.

[13] Sarin SK, Choudhury A, Sharma MK (2019). Acute-on-chronic liver failure: consensus recommendations of the Asian Pacific association for the study of the liver (APASL): an update. Hepatol Int.

[14] (2023). EASL Clinical Practice Guidelines on acute-on-chronic liver failure. J Hepatol.

[15] O'Leary JG, Reddy KR, Garcia-Tsao G (2018). NACSELD acute-on-chronic liver failure (NACSELD-ACLF) score predicts 30-day survival in hospitalized patients with cirrhosis. Hepatology.

[16] Amarapurkar D, Dharod MV, Chandnani M (2015). Acute-on-chronic liver failure: a prospective study to determine the clinical profile, outcome, and factors predicting mortality. Indian J Gastroenterol.

[17] Agrawal S, Duseja A, Gupta T, Dhiman RK, Chawla Y (2015). Simple organ failure count versus CANONIC grading system for predicting mortality in acute-on-chronic liver failure. J Gastroenterol Hepatol.

[18] Khot AA, Somani P, Rathi P, Amarapurkar A (2014). Prognostic factors in acute-on-chronic liver failure: a prospective study from western India. Indian J Gastroenterol.

[19] Pati GK, Singh A, Misra B, Misra D, Das HS, Panda C, Singh SP (2016). Acute-on-chronic liver failure (ACLF) in coastal eastern India: "a single-center experience". J Clin Exp Hepatol.

[20] Rastogi A, Kumar A, Sakhuja P (2011). Liver histology as predictor of outcome in patients with acute-on-chronic liver failure (ACLF). Virchows Arch.

[21] Huang K, Hu JH, Wang HF (2011). Survival and prognostic factors in hepatitis B virus-related acute-on-chronic liver failure. World J Gastroenterol.

[22] Xu Z, Ren X, Liu Y (2011). Association of hepatitis B virus mutations in basal core promoter and precore regions with severity of liver disease: an investigation of 793 Chinese patients with mild and severe chronic hepatitis B and acute-on-chronic liver failure. J Gastroenterol.

[23] Dhiman RK, Agrawal S, Gupta T, Duseja A, Chawla Y (2014). Chronic Liver Failure-Sequential Organ Failure Assessment is better than the Asia-Pacific Association for the Study of Liver criteria for defining acute-on-chronic liver failure and predicting outcome. World J Gastroenterol.

[24] Gustot T, Fernandez J, Garcia E (2015). Clinical course of acute-on-chronic liver failure syndrome and effects on prognosis. Hepatology.

[25] Ferreira Cardoso M, Alexandrino G, Carvalho E Branco J, Anapaz V, Carvalho R, Horta D, Martins A (2019). The impact and evolution of acute-on-chronic liver failure in decompensated cirrhosis: a Portuguese single-center study. Gastroenterol Hepatol.

[26] Arroyo V, Moreau R, Jalan R, Ginès P (2015). Acute-on-chronic liver failure: a new syndrome that will re-classify cirrhosis. J Hepatol.

[27] Poudel SC, Acharya A, Maharjan S, Gc S, Shrestha R, Thapa S, Poudel S (2023). Chronic liver disease among patients admitted in the department of internal medicine of a tertiary care centre: a descriptive cross-sectional study. JNMA J Nepal Med Assoc.

[28] Rathod SD, Luitel NP, Jordans MJ (2018). Prevalence and correlates of alcohol use in a central Nepal district: secondary analysis of a population-based cross-sectional study. Glob Ment Health (Camb).

[29] Shrestha A (2016). Viral hepatitis in Nepal: past, present, and future. Euroasian J Hepatogastroenterol.

[30] Baral P, Shrestha R, Shrestha RN, Banstola D, Prajapati R (2021). A study of height, weight and body mass index in Nepalese. J Gandaki Med Coll-Nepal.

[31] Philips CA, Paramaguru R, Augustine P, Rajesh S, Ahamed R, George T, Padsalgi G (2019). A single-center experience on outcomes of complementary and alternative medicine use among patients with cirrhosis. Hepatol Commun.

[32] Zakareya T, Akl M, Shibl S, El-Mazaly M, Abdel-Razek W (2022). Utility of prognostic scores in predicting short-term mortality in patients with acute-on-chronic liver failure. Egypt Liver J.

[33] Huang PY, Lin YC, Wang CC, Chen CH (2025). Clinical outcomes and predictors in patients with acute on chronic liver failure in southern Taiwan. J Formos Med Assoc.

[34] Moreau R, Jalan R, Gines P (2013). Acute-on-chronic liver failure is a distinct syndrome that develops in patients with acute decompensation of cirrhosis. Gastroenterology.

[35] Valantine B, Sundaray N, Mishra D, Sahu S, Narayan J, Panda BN, Singh A (2021). Predictors of early mortality among patients with acute-on-chronic liver failure. JGH Open.

[36] Barosa R, Roque Ramos L, Patita M, Nunes G, Fonseca J (2017). CLIF-C ACLF score is a better mortality predictor than MELD, MELD-Na and CTP in patients with acute on chronic liver failure admitted to the ward. Rev Esp Enferm Dig.

[37] Leão GS, Lunardi FL, Picon RV, Tovo CV, de Mattos AA, de Mattos ÂZ (2019). Acute-on-chronic liver failure: a comparison of three different diagnostic criteria. Ann Hepatol.

[38] Antunes AG, Teixeira C, Vaz AM (2017). Comparison of the prognostic value of Chronic Liver Failure Consortium scores and traditional models for predicting mortality in patients with cirrhosis. Gastroenterol Hepatol.

